# Speech perception deficits and the effect of envelope-enhanced story listening combined with phonics intervention in pre-readers at risk for dyslexia

**DOI:** 10.3389/fpsyg.2022.1021767

**Published:** 2022-10-28

**Authors:** Femke Vanden Bempt, Shauni Van Herck, Maria Economou, Jolijn Vanderauwera, Maaike Vandermosten, Jan Wouters, Pol Ghesquière

**Affiliations:** ^1^Parenting and Special Education Research Unit, Faculty of Psychology and Educational Sciences, KU Leuven, Leuven, Belgium; ^2^Research Group ExpORL, Department of Neurosciences, KU Leuven, Leuven, Belgium; ^3^Psychological Sciences Research Institute, Université Catholique de Louvain, Louvain-la-Neuve, Belgium; ^4^Institute of Neuroscience, Université Catholique de Louvain, Louvain-la-Neuve, Belgium

**Keywords:** dyslexia, speech perception deficits, auditory intervention, pre-readers, preventive

## Abstract

Developmental dyslexia is considered to be most effectively addressed with preventive phonics-based interventions, including grapheme-phoneme coupling and blending exercises. These intervention types require intact speech perception abilities, given their large focus on exercises with auditorily presented phonemes. Yet some children with (a risk for) dyslexia experience problems in this domain due to a poorer sensitivity to rise times, i.e., rhythmic acoustic cues present in the speech envelope. As a result, the often subtle speech perception problems could potentially constrain an optimal response to phonics-based interventions in at-risk children. The current study therefore aimed (1) to extend existing research by examining the presence of potential speech perception deficits in pre-readers at cognitive risk for dyslexia when compared to typically developing peers and (2) to explore the added value of a preventive auditory intervention for at-risk pre-readers, targeting rise time sensitivity, on speech perception and other reading-related skills. To obtain the first research objective, we longitudinally compared speech-in-noise perception between 28 5-year-old pre-readers with and 30 peers without a cognitive risk for dyslexia during the second half of the third year of kindergarten. The second research objective was addressed by exploring growth in speech perception and other reading-related skills in an independent sample of 62 at-risk 5-year-old pre-readers who all combined a 12-week preventive phonics-based intervention (GraphoGame-Flemish) with an auditory story listening intervention. In half of the sample, story recordings contained artificially enhanced rise times (GG-FL_EE group, *n* = 31), while in the other half, stories remained unprocessed (GG-FL_NE group, *n* = 31; Clinical Trial Number S60962—https://www.uzleuven.be/nl/clinical-trial-center). Results revealed a slower speech-in-noise perception growth in the at-risk compared to the non-at-risk group, due to an emerged deficit at the end of kindergarten. Concerning the auditory intervention effects, both intervention groups showed equal growth in speech-in-noise perception and other reading-related skills, suggesting no boost of envelope-enhanced story listening on top of the effect of combining GraphoGame-Flemish with listening to unprocessed stories. These findings thus provide evidence for a link between speech perception problems and dyslexia, yet do not support the potential of the auditory intervention in its current form.

## Introduction

The slow amplitude modulations in the speech signal contain important rhythmic acoustic cues, e.g., onset rise times, which highly contribute to the development of speech perception abilities and phonological representations (Thomson and Goswami, 2010; Leong and Goswami, 2015; Goswami, 2019). Rise time sensitivity and speech perception abilities are therefore unsurprisingly linked to language and reading development (Goswami et al., 2016; Goswami, 2019). Accordingly, deficits in these skills in individuals with (a risk for) developmental dyslexia have been widely reported, making rise time sensitivity and speech-in-noise perception, next to addressing specific reading skills, interesting targets for reading intervention programs. Longitudinal study designs which shed light on developmental trajectories of speech perception skills in children with or without a risk for dyslexia are however still rare (Boets et al., 2011; Kalashnikova et al., 2019), yet crucial to determine the potential onset of auditory problems in dyslexia and define the optimal timing for (auditory) interventions. The current study therefore alludes to (1) longitudinally compare the development of speech perception skills in pre-readers with or without an elevated cognitive risk for developmental dyslexia and (2) investigate the added value of an auditory intervention, specifically targeting rise time sensitivity in at-risk pre-readers.

Speech is a highly complex acoustic signal containing an enormous amount of temporal and spectral fluctuations. In the temporal domain, the speech envelope, encompassing the rather slow modulations at rates between 2 and 50 Hz in the overall amplitude, is shown to be particularly relevant for successful speech perception (Drullman et al., 1994; Shannon et al., 1995). Further supporting its linguistic relevance, Greenberg et al. (2003) and Leong (2012) showed that certain envelope amplitude modulation frequencies represent different key phonological units of varying grain sizes: stressed (1–3 Hz), or unstressed syllables (4–8 Hz), onset rimes (15–30 Hz), and phonemes (30–50 Hz). Many studies also reported coherence between speech-relevant envelope amplitude modulation patterns and oscillatory activity in the brain, thereby supporting the envelope relevance for speech perception at the neural level. For instance, at rest, in the absence of an incoming speech signal, neural networks fire and recover from firing electrical pulses, resulting in a variety of different oscillation rates across different cell populations, ranging from delta (1–3 Hz), theta (4–8 Hz), alpha (8–12 Hz), beta (15–30 Hz), low gamma (30–50 Hz), and high gamma oscillations (> 60 Hz; Goswami, 2019). When encoding speech, these neural oscillations reset their activity and start firing along with the prosodic (delta), syllabic (theta), onset-rime (beta), and phonemic (low gamma) rates present in the speech envelope (Giraud and Poeppel, 2012; Poeppel, 2014; Leong and Goswami, 2015). This phenomenon, further referred to as neural entrainment, presumably contributes to optimal and efficient speech processing and as a result, language acquisition (Kotz and Schwartze, 2010; Peelle and Davis, 2012; Mai et al., 2016; Riecke et al., 2018). Interestingly, the abovementioned resetting process of the brain waves, initiating neural entrainment, is facilitated by temporal acoustic cues present in the speech envelope, namely onset rise times. Onset rise times, henceforth rise times, are characterized by sudden rises in speech energy and most prominently occur at syllable onsets (Gross et al., 2013). They highly contribute to the perception of speech rhythm (Goswami and Leong, 2013) and guide the neural system to accurately phase-lock to the envelope amplitude modulation patterns, aiding speech segmentation at different grain-size levels (Doelling et al., 2014; Goswami, 2019). Listening to highly rhythmic speech, e.g., child-directed speech or nursery rhymes (Goswami, 2019), generally characterized by steep rise times (Lallier et al., 2017), is therefore thought to benefit speech perception abilities and the development of solid phonological representations in young language learners (Thomson and Goswami, 2010; Leong and Goswami, 2015; Goswami, 2019). Consequently, rise time sensitivity and speech perception have both been unsurprisingly associated with language and reading development, and their associated impairments, i.e., a developmental language disorder (Ziegler et al., 2005; Corriveau et al., 2007; Cumming et al., 2015; Goswami et al., 2016; Goswami, 2019) and developmental dyslexia (Goswami et al., 2016; Goswami, 2019).

Developmental dyslexia is a learning disability characterized by inaccurate and dysfluent word decoding and/or spelling, despite adequate intelligence and sensory abilities, and sufficient instruction (Lyon et al., 2003; American Psychiatric Association, 2014). Although emerging evidence postulates that the etiology of dyslexia could be interpreted within a multiple deficit framework, suggesting that it results from multiple interacting deficits at the cognitive, neurobiological, genetic, and environmental level (Pennington, 2006; van Bergen et al., 2014), decoding problems in dyslexia have been mostly (but thus not always) attributed to underdeveloped phonological representations, resulting in deficient phonological processing abilities (i.e., rapid naming, phonological awareness, and verbal short-term memory; Wagner and Torgesen, 1987), which in turn impede the acquisition of grapheme-to-phoneme correspondences (Snowling, 2000; Ramus et al., 2003; Vellutino et al., 2004). More recently, Goswami (2011) stated in the so-called Temporal Sampling Framework that these underdeveloped phonological representations in dyslexia further stem from reduced neural entrainment at syllabic and prosodic rates in the speech envelope. Although auditory problems are less frequently associated with dyslexia compared to phonological deficits (Ramus et al., 2003), the Temporal Sampling Framework is widely supported by cross-sectional studies reporting atypical neural entrainment at the syllabic and prosodic level, hampered rise time and speech rhythm sensitivity, and speech-in-noise perception deficits in adults and children with and pre-readers at risk for dyslexia (Goswami et al., 2002; Richardson et al., 2004; Hämäläinen et al., 2005, 2012; Abrams et al., 2009; Ziegler et al., 2009; Thomson and Goswami, 2010; Boets et al., 2011; Goswami, 2011; Poelmans et al., 2011; Dole et al., 2012; Law et al., 2014, 2015, 2017; Leong and Goswami, 2014; Calcus et al., 2016, 2018; Molinaro et al., 2016). Prosodic neural entrainment and the abovementioned temporal auditory processing skills measured at the pre-reading phase were also found to predict later literacy development (Law et al., 2017; Vanvooren et al., 2017; Critten et al., 2021; Ríos-López et al., 2022), further supporting the Temporal Sampling Framework rationale. Longitudinal studies specifically comparing growth trajectories between children at risk for dyslexia and typically developing peers regarding auditory processing skills are however scant (Boets et al., 2011; Kalashnikova et al., 2019), but might be relevant to determine the onset of potential auditory processing problems and to define the optimal timing for auditory interventions in dyslexia.

Evidence exists that dyslexia interventions should encompass a phonics-based approach, combining grapheme-phoneme correspondences and phoneme blending exercises (National Institute of Child Health and Human Development, 2000; Ehri et al., 2001; Snowling and Hulme, 2011; Galuschka et al., 2014). Moreover, phonics-based interventions seem to yield larger benefits when delivered to at-risk children in the third year of kindergarten or first grade (before the onset of reading problems) compared to remediation programs at later ages (Wanzek and Vaughn, 2007; Lovett et al., 2017). Yet, despite the promises of preventive phonics interventions, some at-risk children still fail to develop average reading skills (Torgesen, 2001; Zijlstra et al., 2021). The presence of temporal auditory processing deficits experienced by some (but not all) of the non-responders could be a potential explanation for this phenomenon. Phonics-based interventions indeed heavily rely on speech perception abilities, given their focus on exercises with auditorily presented phonemes, e.g., in the case of phoneme blending training. Although currently uninvestigated, disrupted entrainment to the speech envelope, leading to rise time sensitivity, speech perception, and phonological difficulties, could thus constrain an optimal response to such preventive intervention types. Hence, combining an auditory intervention, specifically targeting rise time sensitivity, with a more typically used preventive phonics intervention could offer a solution to this problem. In line with this hypothesis, several studies indeed already addressed the impact of auditory music or speech rhythm interventions in isolation or combined with direct reading training on early reading and associated skills, yielding mixed results (Bhide et al., 2013; Harrison et al., 2018; Patscheke et al., 2019; but see Cancer and Antonietti, 2022 for a review). More recently, Van Hirtum et al. (2021) suggested that auditory interventions for young pre-reading children at risk for dyslexia should comprise listening to so-called envelope-enhanced speech. Envelope enhancement (EE), enhancing the rise times present in the speech envelope, thereby specifically targeting rise time sensitivity, already showed to benefit speech perception in noisy environments in cochlear implant users (Geurts and Wouters, 1999; Koning and Wouters, 2012, 2016). More importantly, Van Hirtum et al. (2021) and Van Hirtum et al. (2019) applied EE to compare speech-in-noise perception in vocoded and natural speech among dyslectic and typically developing readers. Whereas they first showed speech-in-noise perception delays in children and adults with dyslexia compared to typically developing controls in non-enhanced vocoded and natural speech, they subsequently demonstrated an instant closure of the gap when EE was applied in both speech conditions. Based on these observed benefits, Van Herck et al. (2021) explored the impact of a preventive auditory intervention on rise time sensitivity by applying EE to age-appropriate children story recordings, embedded in a tablet-based gaming context (Vanden Bempt et al., 2022). When they combined the auditory EE-intervention with a 12-week tablet-based phonics intervention, i.e., GraphoGame Flemish (GG-FL; Glatz et al., 2021), provided in the second half of the third kindergarten year, pre-readers at cognitive risk for dyslexia showed a head start in rise time sensitivity immediately after the intervention period, compared to (1) at-risk peers who combined GG-FL with listening to non-enhanced stories and (2) an at-risk active control group who combined listening to non-enhanced stories with tablet-based Lego and Playmobil applications. Although the group of children who combined GG-FL with listening to non-enhanced stories showed a larger improvement in reading and associated skills compared to the abovementioned active and a passive control group, receiving no intervention (Vanden Bempt et al., 2021), the presumed boosting effect of the EE-intervention on top of GG-FL regarding speech-in-noise perception, phonological awareness, and letter knowledge, driven by improved rise time sensitivity, currently remained uninvestigated.

The aim of the current study was two-fold. First, given the lack of longitudinal studies investigating growth over time, we aimed to compare the developmental trajectory of auditory processing skills, i.e., speech-in-noise perception in particular, between pre-readers with and without a cognitive risk for dyslexia along a period that is considered most effective for providing reading interventions, i.e., the third year of kindergarten (Wanzek and Vaughn, 2007; Lovett et al., 2017). Given the widely reported cross-sectional speech-in-noise perception problems in adults (Dole et al., 2012) and children with dyslexia (Ziegler et al., 2009; Poelmans et al., 2011; Calcus et al., 2016, 2018) and in at-risk pre-readers who just started the last kindergarten year (Boets et al., 2011), we hypothesized to find a deviant deficit at all time points, but also a slower speech-in-noise perception development in at-risk compared to typically developing children. Second, we aimed to extend the finding of Van Herck et al. (2021) by exploring the added value of the auditory EE intervention on top of phonics instruction with regard to speech-in-noise perception, phonological awareness, and letter knowledge in at-risk children. To this end, we compared the growth of these measures among the two at-risk groups, also included in the study of Van Herck et al. (2021), who received the preventive 12-week tablet-based GG-FL intervention combined with either the envelope-enhanced (GG-FL_EE group) or the non-enhanced (GG-FL_NE group) auditory (story listening) intervention. Given the short-term head start concerning rise time sensitivity in the GG-FL_EE group (Van Herck et al., 2021), we hypothesized to also find greater improvements from pre- to post-intervention with respect to speech-in-noise perception, phonological awareness, and letter knowledge compared to the GG-FL_NE group.

## Materials and methods

### Participants

Following a large-scale school-based screening, fully described in Verwimp et al. (2020), 149 5-year-old kindergarteners (119 children with and 30 children without an elevated cognitive risk for dyslexia) enrolled to participate in a preventive reading intervention study. Children were identified as having a cognitive risk when they performed above the 10th percentile on a task measuring non-verbal reasoning ability (Raven et al., 1984) and below percentile 30 on at least two out of three tasks measuring predictors of later reading ability, namely rapid naming (Van den bos et al., 2002), letter knowledge (Boets et al., 2010; Geurts et al., 2015), and phonological awareness (Boets et al., 2010; Geurts et al., 2015). In addition, participants were only classified as at-risk if they performed below percentile 40 on letter knowledge, in order to maximally benefit from the preventive phonics-based intervention. The group of 30 typically developing children was matched to the at-risk children based on sex, non-verbal reasoning, and educational environment and performed above percentile 40 on all three reading-related skills. All participants were monolingual Dutch-speaking (Flemish) and born in 2013. They were all in the third and last year of kindergarten and followed a minimal schooling period of 20 months. Given that reading instruction in Flanders (Belgium) only starts in first grade, all children were considered pre-reading. Moreover, none of the participants experienced articulatory and/or developmental language problems, neurological deficits, or hearing impairments. The study was approved by the Medical Ethical Committee of University Hospital of Leuven, KU Leuven (report B322201836276) and all parents/caregivers provided verbal and written ethical consent for the participation of their child.

### Study design and procedure

**Figure 1:**
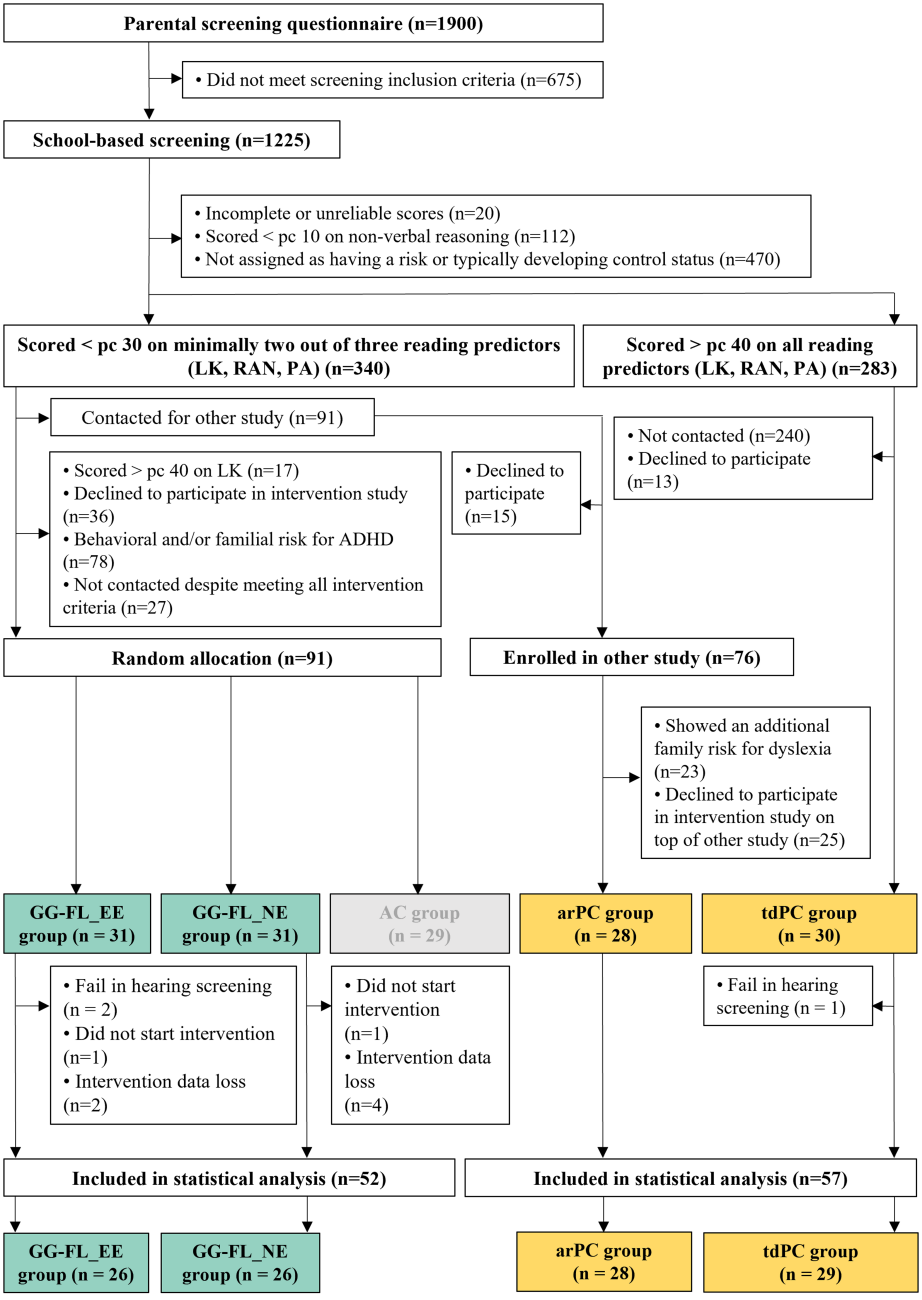
Flowchart from the recruitment and group allocation to data exclusion phase. Yellow groups indicate the groups included for the first study aim. Green groups indicate the groups included for the second study aim. Light grey groups indicate the groups that were not included in the current study. LK, letter knowledge; RAN, rapid naming; PA, phonological awareness; Pc, percentile; ADHD, attention deficit hyperactivity disorder; GG-FL_EE group, GraphoGame Flemish + envelope enhancement group; GG-FL_NE group, GraphoGame Flemish + non-enhanced group; AC group, Active control group; arPC group, at-risk passive control group; and tdPC group, typically developing passive control group.

Ninety-one of the 119 at-risk children that enrolled in the reading intervention project were randomly allocated to one of three experimental groups, using a block randomization procedure taking into account sex, educational environment, and birth trimester: the GG-FL_EE (*n* = 31), the GG-FL_NE (*n* = 31), and the active control (AC) group (*n* = 29). In the block randomization procedure, participants were categorized into different blocks based on their level on each of the three pre-defined categorical factors. Randomization to each of the three experimental groups was then performed within the different blocks, assuring balance regarding these categorical factors across the treatment groups. The GG-FL_EE and GG-FL_NE groups were instructed to train with GG-FL on a tablet at home and to combine this with an auditory tablet-based intervention, which involved listening to either envelope-enhanced or non-enhanced age-appropriate story recordings, respectively (Vanden Bempt et al., 2022). The GG-FL_EE and GG-FL_NE groups are included in the current study in order to disentangle the specific boosting effects of the auditory EE-intervention on speech-in-noise perception and other reading-related skills. The AC group combined the non-enhanced auditory intervention with commercially available tablet-based Lego and Playmobil applications instead of GG-FL. Since this group served as a control group in order to disentangle the specific effects of GG-FL on reading-related skills described in a previous study (Vanden Bempt et al., 2021), the AC group will not be included in the analyses of the current research article. The remaining 28 at-risk children originally enrolled in another ongoing dyslexia study, which recruited 76 non-overlapping at-risk children from the same screening sample. They served as the at-risk passive control group (arPC) in the current study and did not receive reading intervention throughout the project. Given that only three children in the three abovementioned experimental groups (i.e., GG-FL_EE, GG-FL_NE, and AC) had a biological parent with an official dyslexia diagnosis, children in the arPC group were required to only show a “pure” cognitive risk without having an additional family risk. As such, we could eliminate the potential harmful influence of family risk on intervention outcomes (Zijlstra et al., 2021). The 30 typically developing children did also not receive any type of intervention and thus served as the typically developing (tdPC) passive control group. Given the absence of intervention, the arPC and tdPC groups are included in the current study to longitudinally compare speech-in-noise perception between at-risk and non-at-risk pre-readers. Productive letter knowledge and speech-in-noise perception were individually administered at school in a quiet test room in all children at two time points: at the start (pre-test: January–February 2019) and at the end (post-test: April–June 2019) of the second half of the third year of kindergarten. The GG-FL_NE and GG-FL_EE groups received the 12-week tablet-based intervention between these two measurement points (before the COVID-19 pandemic) and received a tablet immediately after the first measurement point at school. Furthermore, next to playing the intervention games for 12 weeks, 6 days per week, 15 min per day at home, the two intervention groups were also instructed to complete five tablet-based assessments of phonological awareness, every 3 weeks, starting from the day after they had received the tablet. Data from 11 children were excluded for statistical analyses, due to (1) a fail in hearing screening in either the first or second measurement phase (*n* = 3), (2) a very poor training integrity by playing less than 10% of both intervention games (*n* = 2), and (3) a loss of auditory intervention data due to technical problems (*n* = 6). As such, group comparisons between the tdPC and arPC group involved 57 participants (arPC: *n* = 28; tdPC: *n* = 29) and group comparisons between the GG-FL_NE and GG-FL_EE group comprised data of 52 participants (26 children in each intervention group). Consider Figure 1 for a complete flowchart of the participant recruitment, group allocation and data exclusion reasons for the whole intervention project. As Van Herck et al. (2021) did not take into account the technical intervention problems experienced by some children during the intervention period, the current intervention groups did not completely (but still almost fully) overlap with the groups included in their study.

### Test battery and questionnaires

#### Speech-in-noise perception

Speech-in-noise perception was administered with the Leuven Intelligibility Peutertest (Lilliput), a speech-in-noise-test suitable for 4–6 year old children (Van Wieringen and Wouters, 2022). Using the software platform APEX (Francart et al., 2008) and test laptops connected to external sound cards (RME Fireface UC interface), seven lists of 11 three-phoneme Dutch consonant-vowel-consonant-words were monaurally presented in speech-weighted noise over a calibrated headphone (Sennheiser HDA-200). Lists were presented to the best functioning ear, which was determined based on a short standardized hearing screening (Guérin and Van Hoeck, 2015), administered prior to the speech-in-noise perception task. All words were recorded by the same female Flemish speaker and in each list, the speech-weighted noise, created based on the average spectrum of the speaker was presented at 65 dB SPL. Yet, the signal-to-noise ratio (SNR) varied among different lists. For each presented list, the child was instructed to repeat each word as accurately as possible. The same first list was always presented at 0 dB SNR and served as a training list in all participants. Then, in accordance with the study of Vanvooren et al. (2017), children listened to random pairs of word lists at −2, −5, and − 8 dB SNR. The maximum raw score in each list was 30, given that the child received one point per correctly repeated phoneme and the first word of each list always served as a training word that did not count in the scoring. For each list, an average percentage of the correctly perceived phonemes was calculated based on the raw scores and ultimately, an average percentage per SNR was obtained. When the child did not obtain average SNR scores above or below 50%, an extra pair of lists was presented at 1 or − 11 dB SNR, respectively. Based on a nonlinear regression fit of a logistic function {model expression: 100/[1 + EXP(4*EstimateSlope*(EstimateSRT-SNR))/100]; Vanvooren et al., 2017}, the speech-reception threshold (SRT), i.e., the level by which the participant understands 50% of the presented phonemes, and its standard error, was calculated for each child. The SRT served as the quantitative outcome measure of speech-in-noise perception in this article, by which a lower value indicated better speech-in-noise perception abilities. Although evidence exists that familiarity of words can influence response accuracy in speech-in-noise tests (Wilson et al., 2010), passive comprehension of the offered words was not tested beforehand because of the three following reasons: (1) the words included in the test were selected based on the so-called target word list for 6-year-old children (Schaerlaekens and Lejaegere, 1999), which offers an overview of the passive vocabulary that kindergarteners should have when finishing kindergarten, (2) the test was scored at the phoneme-level and children were explicitly instructed to repeat everything they heard even when they did not know the word, when they thought the word did not exist, or when they only heard parts of it or even one single phoneme, (3) the test battery schedule was very tight as it also included broader language and domain-general tests (as described in Vanden Bempt et al., 2021), which made it practically unfeasible to also include an extra test-specific word comprehension test.

#### Phonological awareness

Phonological awareness was assessed in the GG-FL_EE and GG-FL_NE groups through a tablet-based game referred to as Diesel-X (Geurts et al., 2015). During this self-administered game-based assessment, participants heard a target word accompanied by its visual animation on the tablet screen and they had to select, out of four alternatives, the animation of the dictated word which contained the same end-phoneme as the given target word. The first two trials provided feedback, whereas the following 10 served as test items without feedback. Hence, the maximum score of this test was 10. Children were instructed to perform five assessments at home, every 3 weeks, starting from the day after they received the tablet at the pre-test phase at school. Both training and test items were the same across all five measurements.

#### Productive letter knowledge

Productive letter knowledge was assessed using a letter-card containing the 16 most frequently used letters in Dutch children books (Boets et al., 2010). The child was asked to produce the correct letter names or sounds. No feedback was provided. Hence, the maximum score of this test was 16.

#### Post-intervention questionnaires

After the intervention period, parents of participants in the GG-FL_EE or GG-FL_NE groups were asked to fill out a post-intervention questionnaire concerning factors that could have influenced the interventional progress and thus, the interpretation of the test outcomes. More specifically, they provided information on the child’s motivation to play both games, the amount of provided encouragement to play both intervention games, the usual playing moment, the amount of tablet exposure for other purposes than the intervention, and the amount of weekly book reading sessions. Moreover, parents also indicated whether they provided explicit reading instruction during the intervention period or whether they explicitly helped their child during the home-based phonological awareness assessments. At the post-test assessment phase at school, children from both groups were also asked to rate both intervention games based on a five-point Likert scale-based smiley-o-meter (Read, 2008) ranging from 1 (a very unhappy smiley) meaning “I did not like the game at all” to 5 (a very happy smiley) meaning “I liked the game very much.”

### Interventions

Following the pre-test session at school, both intervention groups received a tablet (Samsung Galaxy Tab E9.6) with the installed intervention games (GG-FL, Diesel-X, and the envelope-enhanced or non-enhanced auditory intervention, depending on the condition), a calibrated headphone (Audiotechnica ATH M20x), and a parental manual. The manual specifically emphasized that children should perform the interventional games and 5-week game-based assessments of phonological awareness independently without receiving content-related help.

#### GraphoGame Flemish

GraphoGame Flemish (GG-FL), developed based on the existing version of the Netherlands (Glatz, 2018), contained 559 mini-games, which gradually focused on the following reading-related skills: grapheme introduction, auditory and visual discrimination, grapheme-phoneme coupling, phoneme blending and counting, reading, and spelling. Children were instructed to train with GG-FL for 15 min per day, 6 days per week over a period of 12 weeks, which equaled a total of 72 gaming sessions and a total playing time of 1,080 min spread over 84 days (see Glatz et al., 2021 for a more detailed description of GG-FL).

#### Auditory intervention

Participants of both intervention groups were asked to combine GG-FL with a tablet-based auditory intervention, which required active listening to age-appropriate children stories, accompanied by book illustrations. The story listening game was entirely developed and programmed by the research group and offered 72 10-min story listening sessions. Sound levels of all stories were set at 65 dB-A, and calibration was performed in a sound proof booth using speech weighted noise developed based on the average spectra of the different speakers (*n* = 9, all females). Story listening sessions were embedded in an age-appropriate game-based context where players situated in an animated game world in which they could earn coins and buy new avatars and gadgets, when responding correctly to story-related questions (see Vanden Bempt et al., 2022 for a detailed overview of the auditory intervention development and design). EE, which enhances rise times of the speech envelopes, was applied on all story recordings in the GG-FL_EE condition and a technical description and visualization of the EE signal processing algorithm is provided by Van Herck et al. (2021). The research group precisely followed up the schedule compliance, as the tablets daily logged and sent intervention exposure times of both games to a university server. As such, parents of children who did not stick to the intervention scheme were contacted by telephone and encouraged to continue playing.

### Statistical analysis

All data visualizations and analyses were performed in RStudio version 4.0.4 (RStudio Team, 2019; R Core Team, 2021), applying an alpha level of 0.05. The analyses comprised four steps. First, we compared groups (arPC versus tdPC and GG-FL_EE versus GG-FL_NE groups) on their demographic characteristics using Mann–Whitney U, Pearson Chi-Squared, or independent samples *t*-tests. Second, we compared speech-in-noise growth trajectories between the arPC and tdPC groups using linear mixed-effects models (LMMs; lme4 package) (Bates et al., 2015). Timepoint (pre-test vs. post-test) and group (arPC vs. tdPC) were set as fixed categorical within- and between-subjects factors, respectively. The model included by-subject intercepts and a weighing variable, calculated by inversing the standard errors of each individual SRT. As such, participants with less precise speech-in-noise values, i.e., with larger standard errors of the estimated SRT, contributed less to the model estimates than children with more accurate estimates. The third analysis step involved comparing pre-intervention measurements of speech-in-noise perception, phonological awareness, and productive letter knowledge, and intervention-related aspects across the GG-FL_NE and GG-FL_EE groups using Mann–Whitney U, independent samples *t*-tests, Pearson Chi-squared, or Fisher exact tests. Noteworthy, since not all children conducted the first phonological awareness assessment as instructed (i.e., the day after receiving the tablet at school), only measurements of children who completed less than 15% of both interventions (*n* = 46) before conducting the first phonological awareness assessment were included as baseline measurements of this variable. The intervention-related aspects included game exposure and training duration, number of book reading sessions per week, usual playing moment, motivation and encouragement needed to play both games. In the fourth analysis step, we unraveled the potential boosting effect of the auditory EE-intervention on speech-in-noise perception, phonological awareness, and productive letter knowledge by comparing growth differences across the two intervention groups based on LMMs. For LMMs with dependent variables that were only administered twice (speech-in-noise perception and productive letter knowledge), models were constructed in the exact same way as when comparing growth trajectories between the arPC and tdPC group, with the only difference that we did not include a weighing variable in the model where productive letter knowledge was set as the dependent variable. Phonological awareness was modeled as a function of group and training days, given that not all children performed the assessments on the instructed dates (i.e., every 3 weeks for a total of five times starting from the day after they received the tablet) and that some children underwent less or even more than five assessments (maximally seven). Phonological awareness measures from three children were not included in the analysis, as parents reported content-related help to their child during the home-based assessments. Given that the phonological awareness model failed to converge when intercepts and slopes were allowed to vary across subjects, we continued with the random-intercept only model, for which convergence was obtained. For all conducted LMMs in the current study, Type-III analysis of variance tables were calculated using the Satterthwaite approximation for degrees of freedom and estimated marginal means were compared using the emmeans package (Russell, 2021) in case of significant group*time or group*training days interaction effects, indicating significant growth differences between the compared groups. In case of an insignificant interaction effect, two types of follow-up analyses were conducted in order to rule out the possibility that game exposure differences simply masked a possible intervention effect. First, LMMs were repeated with the exclusion of children who behaved as outliers regarding exposure times of either GG-FL and/or the auditory intervention. This resulted in an additional exclusion of one child in the GG-FL_NE group and four children in the GG-FL_EE group. Second, LMMs were repeated with only those children who played at least 80% of the instructed exposure time of both intervention games. This resulted in an additional exclusion of nine children in the GG-FL_NE group and eight children in the GG-FL_EE group.

## Results

### Demographic characteristics

Tables 1, 2 provide an overview of the demographic characteristics of the participants and confirm that the arPC vs. the tdPC groups and the GG-FL_NE vs. the GG-FL_EE groups were matched with respect to age, SES, sex, non-verbal IQ, home literacy environment, and parental reading level. Although research suggests a link between parental reading level and cognitive risk status (Pennington and Lefly, 2001), the insignificant parental reading level differences between the arPC and tdPC group can potentially be explained based on the inclusion criteria of the arPC group.

**Table 1:** Demographic characteristics of the typically developing and at-risk passive control group.

	tdPC				arPC				Statistic_(*df*)_	*p*
	*n*	*M*	*SD*	*Med*	*n*	*M*	*SD*	*Med*		
Age in months at pre-test	29	67.59	3.53	67.00	28	67.43	3.51	68.00	*U* = 396.50	0.879^b^
Age in moths at post-test	29	70.83	3.62	71.00	28	70.50	3.39	71.00	*t*_(55)_ = −0.35	0.726^c^
Non-verbal IQ^a^	29	103.42	15.95	101.75	28	94.92	14.26	93.44	U = 283.50	0.050^b^
HLE (frequency of joint literate activities)^a^	26	0.31	0.85	0.38	28	0.30	0.83	0.29	*t*_(52)_ = −0.05	0.959^c^
HLE (number of books at home)^e^	26	−0.34	1.31	−0.36	28	0.05	0.83	0.19	*U =* 314.00	0.387^b^
HLE (duration and frequency of reading)^e^	26	−0.06	0.85	−0.39	28	0.09	0.97	−0.40	*U =* 344.00	0.729^b^
HLE (duration of joint literate activities)^e^	26	−0.28	0.88	−0.73	28	0.25	1.03	0.26	*U =* 327.00	0.522^b^
Maternal ARHQ score^e^	26	−0.11	0.76	−0.18	28	−0.14	0.61	−0.10	*t*_(52)_ = −0.18	0.858^c^
Paternal ARHQ score^e^	22	−0.46	0.73	−0.54	28	−0.24	0.49	−0.08	*U =* 244.00	0.211^b^
Sex (female/male)	29	16/13			28	17/11			*χ^2^*_(1)_ = 0.18	0.672^d^
SES (low/middle/high)	29	4/10/15			28	7/14/7			*χ^2^*_(2)_ = 4.38	0.112^d^

tdPC, Typically developing passive control group; arPC, at-risk passive control group; df, degrees of freedom; IQ, intelligence quotient; HLE, home literacy environment; ARHQ, adult reading history questionnaire; SES, socio-economic status; n, number of participants; M, mean; SD, standard deviation; and Med, median. For categorical variables (sex and SES), the number of children in each subcategory is reported. Given that not all parents completed the demographic questionnaire during the screening phase, HLE and ARHQ scores contain missing values.

a Standardized scores (M = 100, SD = 15).

b Independent-Samples Mann Whitney U test.

c Independent-Samples *t*-test.

d Pearson Chi-Square test.

e Standardized factor scores based on parental screening questionnaires.

**Table 2:** Demographic characteristics of the GG-FL_NE and GG-FL_EE group.

	GG-FL_NE				GG-FL_EE				Statistic_(*df*)_	*p*
	*n*	*M*	*SD*	*Med*	*n*	*M*	*SD*	*Med*		
Age in months at pre-test	26	65.62	3.32	65.00	26	66.19	3.27	66.50	*U* = 302.50	0.514^b^
Age in moths at post-test	26	69.12	3.44	68.50	26	69.77	3.29	70.00	*U = 291.00*	0.387^b^
Non-verbal IQ^a^	26	102.36	17.68	102.94	26	101.74	14.18	101.25	*t*_(50)_ = −0.140	0.890^c^
HLE (frequency of joint literate activities)^a^	25	−0.20	0.94	−0.17	24	−0.02	0.80	0.26	*U =* 270.00	0.548^b^
HLE (number of books at home)^e^	25	0.23	1.14	0.74	24	0.14	1.11	0.49	*U =* 253.50	0.352^b^
HLE (duration and frequency of reading)^e^	25	−0.15	0.88	−0.49	24	−0.29	0.65	−0.47	*U =* 286.50	0.787^b^
HLE (duration of joint literate activities)^e^	25	−0.14	0.97	−0.77	24	0.15	1.03	−0.71	*U =* 220.00	0.110^b^
Maternal ARHQ score^e^	26	0.11	0.90	−0.13	24	0.17	1.03	−0.06	*t*_(48)_ = 0.226	0.822^c^
Paternal ARHQ score^e^	18	0.34	1.37	0.21	18	0.30	0.88	0.52	*U =* 161.50	0.987^b^
Sex (female/male)	26	14/12			26	10/16			*χ^2^*_(1)_ = 1.24	0.266^d^
SES (low/middle/high)	26	7/10/9			26	8/8/10			*χ^2^*_(2)_ = 0.342	0.843^d^

GG-FL_NE, GraphoGame Flemish + non-enhanced group; GG-FL_EE, GraphoGame Flemish + envelope enhancement group; df, degrees of freedom; IQ, intelligence quotient; HLE, home literacy environment; ARHQ, adult reading history questionnaire; SES, socio-economic status; *n*, number of participants; M, mean; SD, standard deviation; and Med, median. For categorical variables (sex and SES), the number of children in each subcategory is reported. Given that not all parents completed the demographic questionnaire during the screening phase, HLE and ARHQ scores contain missing values.

a Standardized scores (M = 100, SD = 15).

b Independent-Samples Mann Whitney U test.

c Independent-samples *t*-test.

d Pearson Chi-Square test.

e Standardized factor scores based on parental screening questionnaires.

### Speech-in-noise perception growth trajectories in the arPC vs. tdPC groups

**Figure 2:**
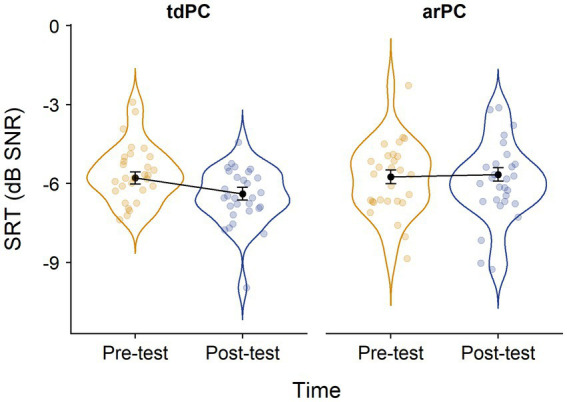
Distribution of the raw SRT values across groups and timepoints. SRT, speech reception threshold; tdPC, typically developing passive control group; and arPC, at-risk passive control group. Black dots and error bars represent the model estimates and their standard errors, respectively. Lower SRTs mean better speech-in-noise perception skills.

Figure 2 shows the distribution of the raw SRT values and the model estimates of the weighed LMM across the arPC and tdPC groups and timepoints. Model outputs revealed no significant main effect of time [*F*_(1,80.421)_ = 2.19, *p* = 0.143] and no significant main effect of group [*F*_(1,59.09)_ = 1.64, *p* = 0.205]. Yet, the group*time interaction was significant [*F*_(1,80.421)_ = 4.07, *p* = 0.047]. *Post-hoc* independent samples *t*-tests showed that at pre-test, groups did not differ significantly (Estimated difference = − 0.04, SE_*estimated difference*_ = 0.35, *t*_(225)_ = − 0.115, *p* = 0.908), whereas at post-test, a significant difference in favor of the tdPC group was found (Estimated difference = − 0.73, SE_*estimated difference*_ = 0.35, *t*_(221)_ = − 2.08, *p* = 0.039), indicating a subtle speech-in-noise perception problem in the at-risk children which emerged during the second half of the third year of kindergarten. *Post-hoc* dependent samples *t*-tests also showed a significant speech-in-noise improvement over time in the tdPC group (Estimated difference = 0.60, SE_*estimated difference*_ = 0.22, *t*_(632)_ = 2.799, *p* = 0.005), which was absent in the arPC group (Estimated difference = −0.09, SE_*estimated difference*_ = 0.27, *t*_(264)_ = −0.340, *p* = 0.734).

### Pre-intervention measurements and intervention-related aspects

**Figure 3:**
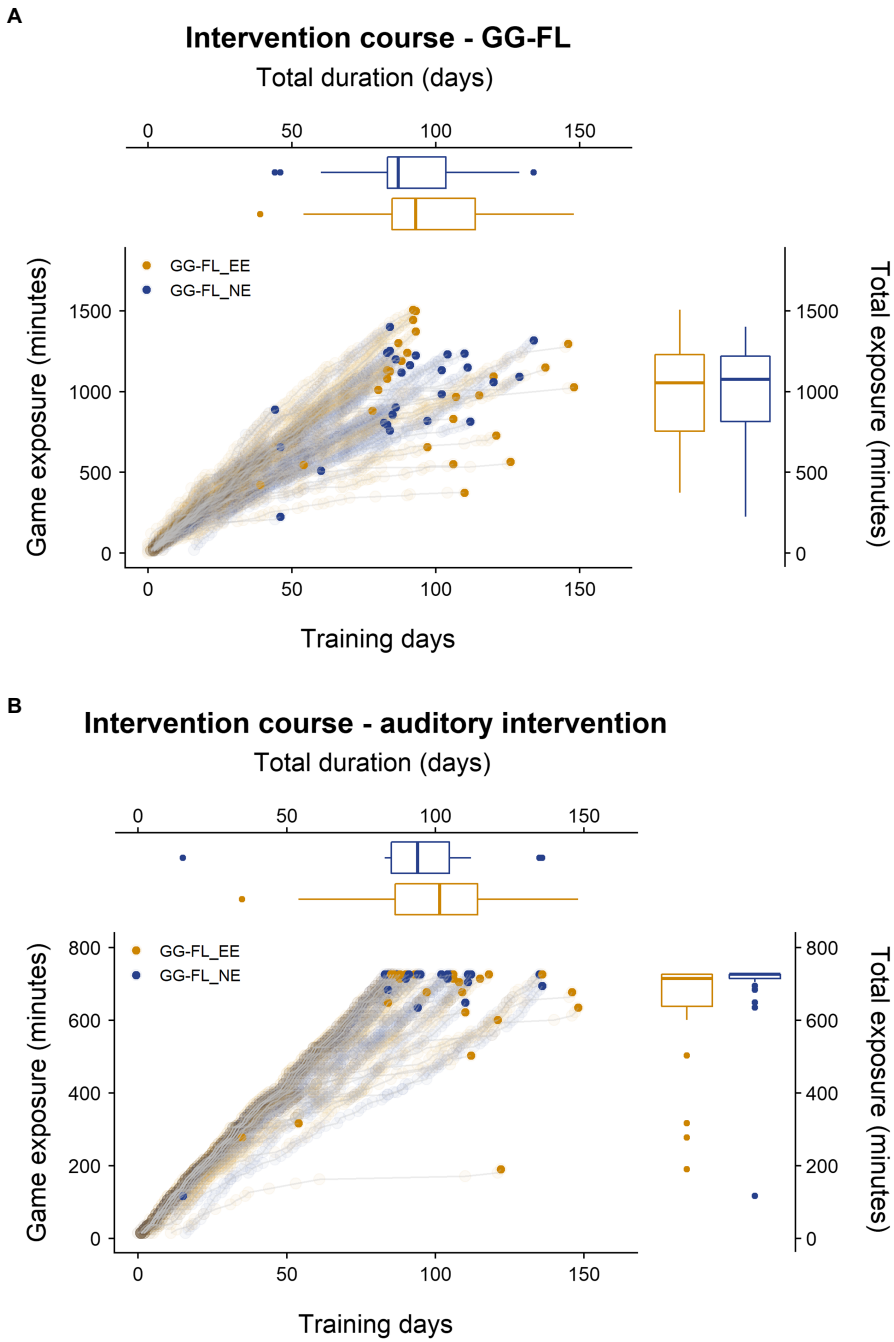
Individual intervention trajectories and the distribution of the final intervention exposure (minutes) and final training duration (days). **(A)** Trajectories of GG-FL. **(B)** Trajectories of the auditory intervention. Bold dots represent individuals’ final game exposures and final training durations. GG-FL, GraphoGame Flemish; GG-FL_EE, GraphoGame Flemish + envelope enhancement group; and GG-FL_NE, GraphoGame Flemish + non-enhanced group.

Mann Whitney U tests revealed no significant differences at baseline between the two intervention groups for speech-in-noise perception (*U* = 332.00, *p* = 0.913), phonological awareness (*U* = 231.00, *p* = 0.462), and productive letter knowledge (*U* = 309.00, *p* = 0.589). Concerning intervention-related aspects, Figure 3 confirms that the majority of the children (nearly) reached the required exposure time, although it often took longer than 84 days. A Mann–Whitney U test revealed that both groups played an equal amount of GG-FL (*U* = 333.00, *p* = 0.927). Yet, concerning the auditory intervention exposure, the GG-FL_EE group listened to significantly fewer stories than the GG-FL_NE group did (*U* = 232.50, *p* = 0.034). Training duration did not differ significantly across groups for both games (GG-FL: *U* = 273.50, *p* = 0.237—auditory intervention: *U* = 278.00, *p* = 0.272). Table 3 shows the outcomes of the remaining intervention-related factors, obtained by the parental post-intervention questionnaires, across the two groups. Apart from the encouragement needed to play GG-FL, by which the GG_FL_EE group needed significantly more parental stimulation to start playing, groups were comparable concerning factors that could have influenced the intervention process.

**Table 3:** Group comparisons of intervention-related factors.

	GG-FL_NE					GG-FL_EE					*Statistic*	*p*
	*n*	*M*	*SD*	*Med*	*IQR*	*n*	*M*	*SD*	*Med*	*IQR*		
Extra tablet exposure (minutes/week)	21	85.71	124.88	0.00	165.00	20	57.00	120.36	0.00	30.00	*U =* 172.00	0.259^a^
Motivation (GG-FL) reported by child (five-point Likert scale)	26	2.85	1.54	2.50	3.00	26	3.00	1.47	3.00	3.00	*U =* 316.00	0.678^a^
Motivation (auditory intervention) reported by child (five-point Likert scale)	26	4.69	0.68	5.00	0.00	26	4.54	0.81	5.00	1.00	*U =* 310.00	0.486^a^
Motivation (GG-FL) reported by parent (VM/RM/ID/NM/unknown)	26	4/13/2/5/0/2				26	2/8/2/6/1/7				-	0.336^b^
Motivation (auditory intervention) reported by parent (VM/RM/ID/LM/NM/unknown)	26	17/9/0/0/0/0				26	9/12/1/1/1/2				-	0.075^b^
Encouragement to play GG-FL (ENN/ESN/EMN/EAN/unknown)	26	2/16/3/3/2				26	2/6/5/12/1				-	0.016^b^
Encouragement to play auditory intervention (ENN/ESN/EMN/EAN/unknown)	26	17/8/1/0/0				26	11/8/4/2/1				-	0.173^b^
Usual playing moment of GG-FL (MN/A/E/V/unknown)	26	4/3/12/6/1				26	2/4/16/2/2				-	0.483^b^
Usual playing moment of the auditory intervention (MN/N/A/E/V/unknown)	26	1/1/18/6/0				26	0/2/21/2/1				-	0.401^b^
Book reading sessions during intervention period (never/rarely/once a week/ multiple times per week/daily/unknown)	26	9/1/2/8/5/1				26	7/0/2/9/5/3				-	0.921^b^

GG-FL_NE, GraphoGame Flemish + non-enhanced group; GG-FL_EE, GraphoGame Flemish + envelope enhancement group; *n*, number of participants; M, mean; SD, standard deviation; Med, median; and IQR, interquartile range. For categorical variables (motivation reported by parent, encouragement to play, usual playing moment, and book reading sessions), the number of children in each subcategory is reported. Given that not all parents completed the full post-intervention questionnaire, extra tablet exposure (minutes/week) contains missing values. VM, very motivated; RM, relatively motivated; ID, independent; NM, not motivated; ENN, encouragement never needed; ESN, encouragement sometimes needed; EMN, encouragement mostly needed; EAN, encouragement always needed; MN, morning; N, noon; A, afternoon; E, evening; and V, varying playing moment.

a Independent-Samples Mann–Whitney U test.

b Fisher’s Exact test.

### Boosting effect of the auditory EE-intervention

**Figure 4:**
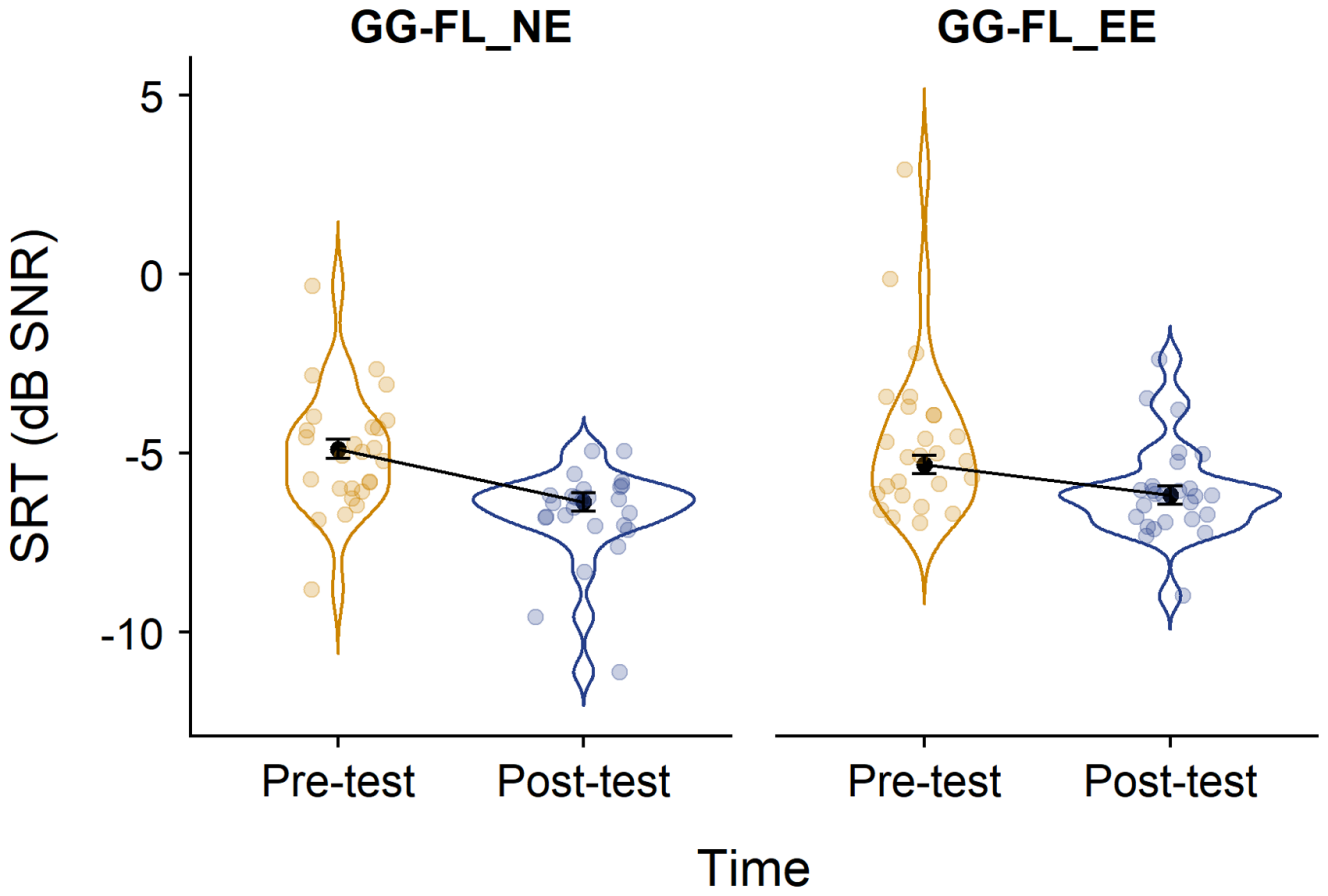
Distribution of the raw SRT values across groups and timepoints. SRT, speech reception threshold; GG-FL_EE, GraphoGame Flemish + envelope enhancement group; and GG-FL_NE, GraphoGame Flemish + non-enhanced group. Black dots and error bars represent the model estimates and their standard errors, respectively.

Figure 4 shows the distribution of the raw SRT values and the model estimates of the weighed LMM across the GG-FL_NE and GG-FL_EE groups and timepoints. The weighed LMM revealed a significant main effect of timepoint [*F*_(1,54.94)_ = 44.00, *p* < 0.001], but no significant main effect of group [*F*_(1,37.68)_ = 0.14, *p* = 0.706]. The interaction effect between group and timepoints also remained insignificant [*F*_(1,54.94)_ = 3.02, *p* = 0.088]. Follow-up analyses with the exclusion of children who behaved as outliers concerning the exposure times of GG-FL and/or the auditory intervention yielded the same pattern of results: a significant main effect of timepoint [*F*_(1,42.13)_ = 44.22, *p* < 0.001] and insignificant group [*F*_(1,25.55)_ = 1.39, *p* = 0.249] and group*timepoint [*F*_(1,42.13)_ = 2.61, *p* = 0.113] effects. The same held true for the LMMs with the exclusion of children who obtained less than 80% of the instructed exposure time of one of the two games: a significant main effect of timepoint [*F*_(1,33.42)_ = 44.09, *p* < 0.001], but insignificant group [*F*_(1,22.62)_ = 1.54, *p* = 0.228] and group*timepoint [*F*_(1,33.42)_ = 1.47, *p* = 0.233] effects. Overall these findings indicate a general speech-in-noise improvement over time, without a significant contribution of listening to envelope-enhanced stories.

**Figure 5:**
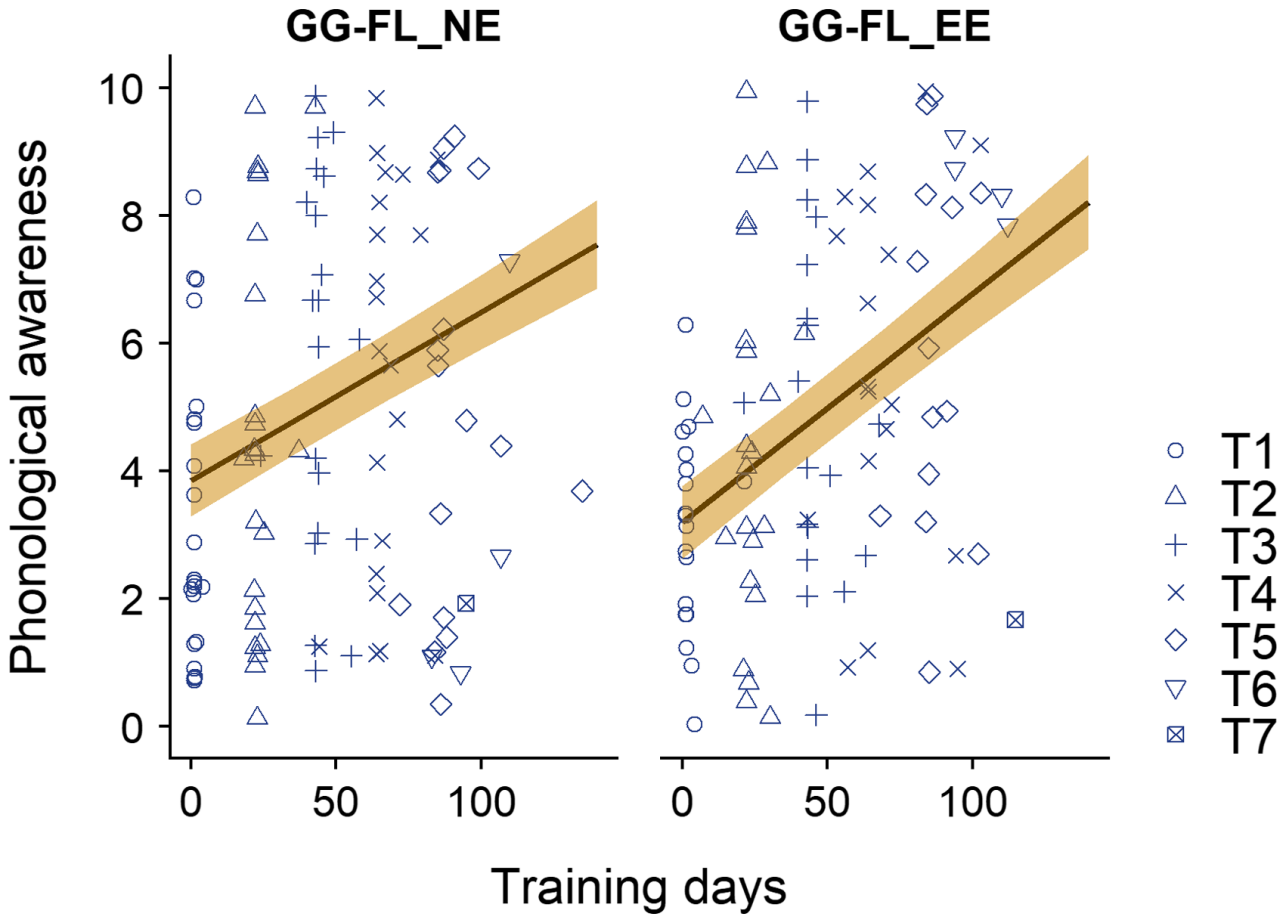
Raw phonological awareness scores across the intermediate assessments and groups. GG-FL_EE, GraphoGame Flemish + envelope enhancement group; GG-FL_NE, GraphoGame Flemish + non-enhanced group; and T1-T7, trial 1–7. The solid lines and their yellow shades represent the estimated linear model slopes and their standard errors.

Concerning phonological awareness, for which the raw scores and model estimates across groups and assessments are represented in Figure 5, the LMM revealed a main effect of training days [*F*_(1,173.19)_ = 76.95, *p* < 0.001]. Yet, the main effect of group [*F*_(1,63.53)_ = 0.67, *p* = 0.417] and the interaction effect between training days and group [*F*_(1,173.19)_ = 1.76, *p* = 0.186] did not reach significance. The same pattern of results was found for the follow-up analysis where outliers in either one of the two or both game exposure times were excluded. The main effect of training days was significant [*F*_(1,166.95)_ = 79.16, *p* < 0.001], while the main effect of group [*F*_(1,56.92)_ = 0.54, *p* = 0.464] and the interaction effect of group and training days [*F*_(1,166.95)_ = 2.24, *p* = 0.136] remained insignificant. The follow-up analyses where only children who played at least 80% of the instructed exposure of both intervention games were included yielded the same trend of outcomes: a significant main effect of training days [*F*_(1,135.39)_ = 57.22, *p* < 0.001], and insignificant group [*F*_(1,46.35)_ = 0.00, *p* = 0.995] and group*training days effects [*F*_(1,135.39)_ = 1.31, *p* = 0.256].

**Figure 6:**
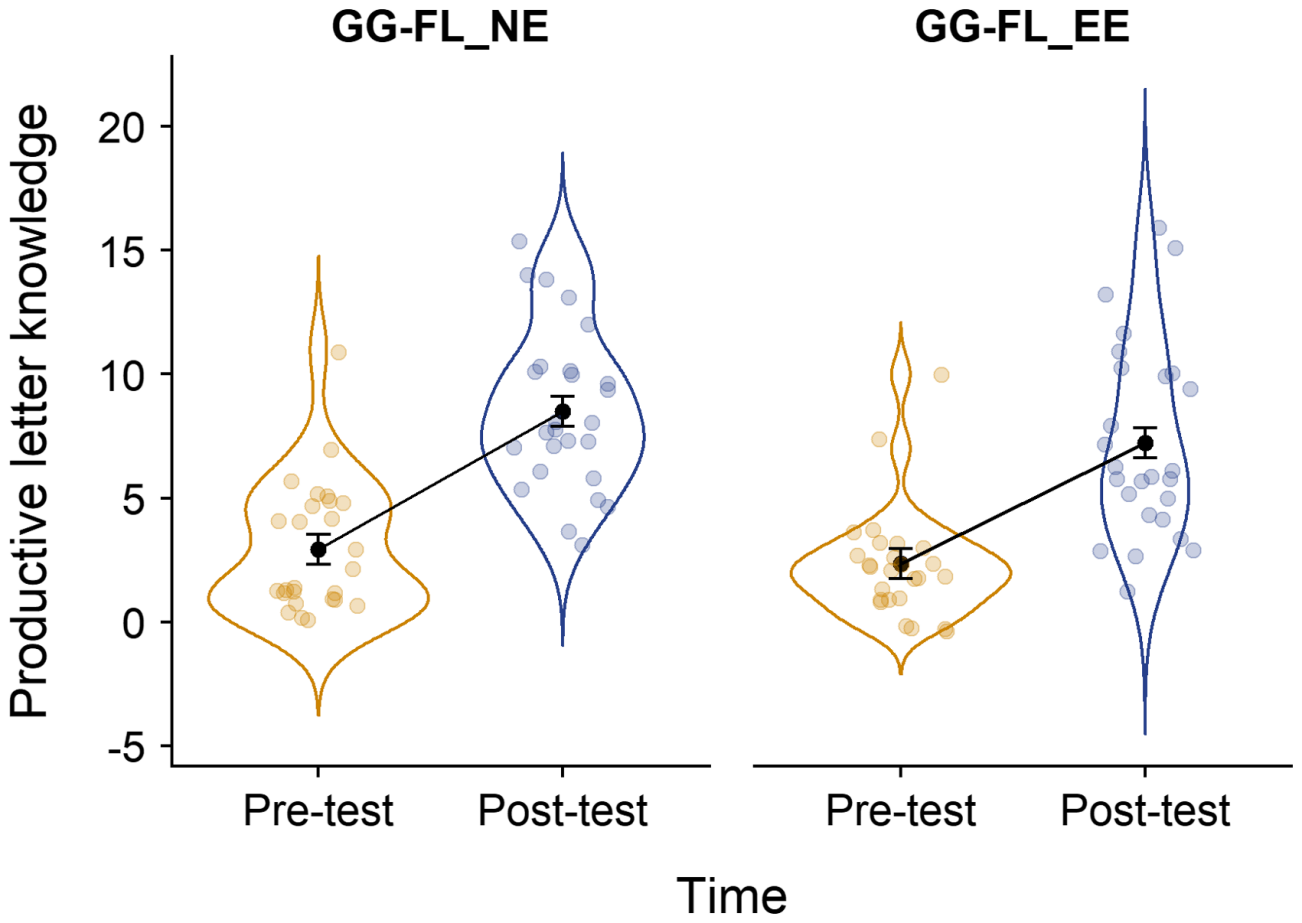
Distribution of the raw productive letter knowledge scores across groups and timepoints. LK, letter knowledge; GG-FL_EE, GraphoGame Flemish + envelope enhancement group; and GG-FL_NE, GraphoGame Flemish + non-enhanced group. Black dots and error bars represent the model estimates and their standard errors, respectively.

For productive letter knowledge (see Figure 6 for the raw scores and model estimates across groups and timepoints), the LMM also revealed a significant main effect of timepoint [*F*_(1,50)_ = 169.78, *p* < 0.001], but an insignificant main effect of group [*F*_(1,50)_ = 1.51, *p* = 0.224] and an insignificant interaction effect between group and timepoint [*F*_(1,50)_ = 0.74, *p* = 0.393]. Follow-up analyses with the exclusion of outliers in the exposure times of GG-FL and/or the auditory intervention yielded the same pattern of results: a significant main effect of timepoint [*F*_(1,45)_ = 171.09, *p* < 0.001] and insignificant group [*F*_(1,45)_ = 0.53, *p* = 0.472] and group*timepoint [*F*_(1,45)_ = 0.01, *p* = 0.927] effects. Moreover, follow-up analyses with the exclusion of children who played less than 80% of the instructed game exposure of one of the two interventions yielded the same pattern: a significant main effect of timepoint [*F*_(1.33)_ = 133.08, *p* < 0.001], but insignificant group [*F*_(1,33)_ = 0.08, *p* = 0.774] and group*timepoint [*F*_(1,33)_ = 0.003, *p* = 0.987] effects. Hence, as for speech-in-noise perception, the results show overall growth over time, but no indication of a boosting effect of listening to envelope-enhanced speech on phonological awareness and productive letter knowledge.

## Discussion

The current study aimed at (1) comparing the development of speech-in-noise perception between pre-readers with or without a cognitive risk for dyslexia during one of the most effective periods for (phonics-based) reading interventions and (2) exploring the presence of a boosting effect on speech-in-noise perception, phonological awareness, and productive letter knowledge of listening to envelope-enhanced speech on top of receiving phonics instruction in at-risk children. To achieve the first aim, we compared the growth in speech-in-noise perception among at-risk and typically developing pre-readers who did not receive any type of preventive reading intervention during the second half of the third year of kindergarten. The results revealed a slower speech-in-noise perception development in the arPC compared to the tdPC group, due to poorer speech-in-noise-perception performance of the arPC group at the second, but not at the first measurement point. Regarding the second research aim of the current study, we included largely overlapping groups from the study of Van Herck et al. (2021), which revealed an EE-driven intervention effect on rise time sensitivity, and compared the growth in speech-in-noise perception, phonological awareness, and letter knowledge of children who received a preventive phonics-based intervention, GG-FL, combined with listening to either envelope-enhanced (GG-FL_EE group) or non-enhanced stories (GG-FL_NE group). The equal growth concerning all three abovementioned reading-related variables across both intervention groups indicates that listening to envelope-enhanced speech did not promote literacy development more than the phonics-based intervention combined with listening to unprocessed stories did. Yet, despite not directly compared with the active control group in the current study, our findings, together with the results reported in Vanden Bempt et al. (2021), do provide good support for the added value of offering GG-FL to at-risk kindergarteners in the pre-reading stage.

The slower development of speech-in-noise perception and the worse performance at the second measurement point in the group of at-risk pre-readers compared to their typically developing peers fits within the Temporal Sampling Framework proposed by Goswami (2011). These findings could therefore explain why preventive phonics-based interventions are not optimally effective for all at-risk children (Torgesen, 2001), given that auditory processing deficits could possibly hinder the development of phonological awareness and reading when exercises are provided solely in the auditory modality (e.g., phoneme blending). Contrary to our hypothesis and not in line with other studies that demonstrated speech perception deficits in at-risk children already at the start of the last kindergarten year (Boets et al., 2011), our results show that the speech-in-noise perception deficit only emerged during the second half of the school year. This suggests that the onset of speech perception deficits in dyslexia is dynamic and individual in nature rather than fixed and emphasizes the need for acquiring longitudinal data in order to define individually-based deficit onsets and optimal timing for intervention. Concerning optimal intervention timing, based on our group results, we hypothesize that auditory interventions in dyslexia, unlike phonics-based programs (Wanzek and Vaughn, 2007; Lovett et al., 2017), are best provided even before the third year of kindergarten, before the possible onset of speech perception deficits. Although this remains to be fully investigated based on randomized controlled trials (RCT) and future meta-analyses, this hypothesis would have even gained more support if the same developmental pattern would have been observed with respect to rise time sensitivity. Yet, as Van Herck et al. (2021) did not administer rise time sensitivity in the group of typically developing children, this hypothesis could not be explored any further.

Regarding the absence of a boosting effect of the auditory EE-intervention, five putatively important explanations are discussed below. The first and most plausible one relates to the training-specificity of the auditory EE-intervention. More specifically, it is noteworthy that Van Herck et al. (2021) did find a short-term EE-driven boosting effect on rise time sensitivity in our sample. Possibly, given the focus on enhanced rise times, the specific auditory EE-intervention was able to directly train rise time sensitivity, without yielding transfer effects on skills that were not directly targeted (speech-in-noise perception, phonological awareness, and productive letter knowledge). These findings correspond to other preventive though phonics-based reading interventions, which failed to obtain transfer effects on indirectly trained phonological or broader language skills (Schneider et al., 2000; Elbro and Petersen, 2004; Regtvoort and Van Der Leij, 2007; Bowyer-Crane et al., 2008; Van Otterloo and Van Der Leij, 2009). As hypothesized by Vanden Bempt et al. (2021), reading interventions in young pre-reading age groups, such as the ones described in the current study, seem rather training-specific. A second explanation for the null-effects could relate to the intervention exposure. It is possible that the EE-intervention was actually able to boost auditory and reading-related skills, but that its effects were masked because the GG-FL_EE group simply listened to fewer stories than the GG-FL_NE group did. The question is now why this phenomenon occurred. Possibly, listening to envelope-enhanced stories might have been experienced as less pleasant given the sudden rises in speech energy at syllable onsets, decreasing exposure times. However, previous experiments showed no differences in loudness perception when comparing listening to envelope-enhanced speech with listening to unprocessed natural speech (Koning and Wouters, 2016; Van Hirtum et al., 2019), raising the idea that other factors contributed to the observed game exposure difference. Alternatively, the reported higher encouragement needed to play GG-FL in the GG-FL_EE compared to the GG-FL_NE group could simply point to a generally lower tablet play motivation in the former group, which could have affected exposure times (Sweetser and Wyeth, 2005). However, as we were also unable to find auditory intervention-driven effects nor in the subsample of children which did not behave as outliers regarding intervention exposure times nor in the subsample that obtained at least 80% of the instructed exposure time of both games, the hypothesis that game exposure differences masked possible intervention effects is rather unplausible. A third explanation for the absence of the specific EE-effect could relate to the timing of the auditory EE-intervention. More specifically, evidence exists that the development of phonological representations already emerges during the first year of life. Infants then gradually develop a language-specific listening pattern, characterized by a decline of discriminating non-native and an improvement in distinguishing native-language phonetic contrasts (Werker and Tees, 1984; Kuhl et al., 2006). Behavioral and neural indications of altered phonemic representations have also already been observed in infants at family risk for dyslexia (Guttorm et al., 2001; Richardson et al., 2003; Van Leeuwen et al., 2007; Leppänen et al., 2010; Van Zuijen et al., 2012, 2013; Mittag et al., 2022). These findings suggest that hampered phonological representations, possibly caused by disrupted neural entrainment, already exhibit in the very early stages of development and that the current auditory EE-intervention was simply delivered too late. A fourth explanatory factor for the absence of a boosting EE-effect might relate to the postulated intervention eligibility criteria following the screening procedure. More specifically, as mentioned in the introduction section, developmental dyslexia is nowadays considered as a heterogeneous disorder which results from interacting deficits at the genetic, neurobiological, cognitive, and environmental level (Pennington, 2006). This means that poor readers can develop similar reading profiles caused by different underlying cognitive deficits (Lallier et al., 2017; McArthur and Castles, 2017). Although contradicted (Leppänen et al., 2010), not all at-risk children who will eventually develop reading problems thus experience temporal auditory processing deficits in the pre-reading stage. The screening protocol for the current intervention study only included assessments of the three most robust predictors of later reading ability, namely rapid naming, letter knowledge, and phonological awareness (Catts et al., 2001; Van Der Leij, 2013), without including measures of rise time sensitivity or speech rhythm discrimination. As a result, we probably also included at-risk children without auditory processing deficits, for whom an extra auditory intervention focusing on rise time sensitivity on top of phonics instruction did not provide added value, neutralizing the potential EE-effects at the group-level. Our screening inclusion criteria could also easily explain why we did find behavioral evidence for the effectiveness of GG-FL as reported in Vanden Bempt et al. (2021), since all of the included children performed weak on at least one measure of phonological processing (i.e., phonological awareness or rapid naming). Future studies should include existing age-appropriate rise time sensitivity tasks (e.g., Van Herck et al., 2021) or speech rhythm sensitivity tasks (e.g., Critten et al., 2021) in their screening protocol in order to provide appropriate targeted interventions which fit the cognitive profiles of the population, an approach which is also recommended by Munro (2017). A last explanation for the absence of the boosting effect possibly relates to the acoustic nature of the story recordings. More specifically, in the story recording phase of the envelope-enhanced/non-enhanced auditory intervention development, speakers were explained to read the book texts using a standard Dutch accent and intonation, whereas they were not explicitly instructed to make use of child-directed speech. Compared to adult-directed speech, child-directed speech contains simplified syntactic and semantic features (Soderstrom et al., 2008), occurs at a slower tempo (Fernald and Simon, 1984), with a higher pitch (Kuhl et al., 1997), and with more clear-cut intonation patterns (Fernald and Simon, 1984), all of which are causally related to the development of an infant’s phonological system (Liu et al., 2003; Leong and Goswami, 2015). Although not analyzed and compared with adult speech samples, the acoustic features of our envelope-enhanced speech offered to the GG-FL_EE group probably resembled those present in adult-directed speech with the only difference that it contained artificially enhanced onset rise times. “Simply” adding EE on adult-directed speech was possibly not strong enough to robustly improve speech-in-noise perception and sharpen phonological representations on top of phonics intervention in our population and the presence of other child-directed-speech-related features could have possibly impacted reading development more than the current version of the auditory EE-intervention did. The only solution to confirm or reject the abovementioned hypothesis is to conduct a randomized controlled trial comparing the boosting effect of EE applied on story recordings based on adult-directed or child-directed speech.

A last noteworthy discussion point relates to the speech-in-noise development in all at-risk groups included in the current study. Although not statistically compared in the same model, we found speech-in-noise perception improvements from pre- to post-test in the GG-FL_EE and GG-FL_NE groups, but not in the matched arPC group. The growth in speech-in-noise perception in the two intervention groups, together with their equal growth in phonological awareness and productive letter knowledge, might therefore reflect either (1) a specific GG-FL-driven effect consistent with the findings of Vanden Bempt et al. (2021), (2) an effect of general story listening irrespective of envelope enhancement, or (3) a combined effect of both interventions. The possible specific GG-FL-driven effect is based on the hypothesis that learning to read, which was attempted in the GG-FL training, indirectly influences speech-in-noise perception and its neurophysiological correlates, e.g., prosodic neural entrainment (Vanthornhout et al., 2018), *via* its direct positive effect on lexical knowledge (Destoky et al., 2020, 2022). More specifically, reading experience is thought to enlarge vocabulary size (Cain and Oakhill, 2011), which in turn facilitates the development of categorical phoneme perception in a top-down matter, and as a result speech-in-noise perception (McClelland et al., 2006). Nevertheless, the hypothesis that GG-FL drove speech-in-noise perception on its own is relatively unplausible. As it happens, a previous study by Vanden Bempt et al. (2021) reported positive GG-FL-driven word decoding effects in our GG-FL_NE group, yet at a very rudimental level, by which only three children were able to read more than five words. This finding almost leaves out the possibility that reading acquisition *per se* could have had the chance to alter vocabulary size in the two GG-FL groups. Alternatively, regarding the possible effect of envelope-enhanced-independent story listening, research showed that listening to stories in noise as part of a 12-week auditory computer-based intervention aided speech-in-noise perception in school-aged children with auditory processing disorders (Loo et al., 2016), supporting the possibility that our auditory intervention might also have been able to boost speech-in-noise perception in the two intervention samples. Unfortunately, the research design applied in the current study does not allow us to disentangle the specific effects of GG-FL and general story listening, as these aspects were present in both intervention groups. The specific effects of GG-FL were already unraveled in a previous study by comparing growth in reading-related skills in the GG-FL_NE group with an active control group that combined (unprocessed) story listening with Lego-and Playmobil games (Vanden Bempt et al., 2021). Future research should however also include a group that is training with GG-FL and another game that does not involve story listening in order to disentangle general story listening effects on speech perception and reading-related skills.

As in any other study, this study contains several limitations. The first important one relates to the assessment procedure of phonological awareness, which was repeatedly conducted at home five times based on the same training and test items and without supervisory control. Repeatedly offering test items along a short time span, i.e., 3 weeks, could have resulted in practice (and even ceiling) effects and hence an overestimation of the participants’ phonological awareness abilities. Figure 5 indeed suggests ceiling effects of end phoneme identification at the fifth assessment session (T5), the last session for the majority of our participants, in a subsample of the GG-FL_NE, but not in the GG-FL_EE group. Moreover, as stated in Vanden Bempt et al. (2021), the lack of supervision might have resulted in less reliable measurements due to (1) distraction, (2) forbidden provided content-related help by parents or siblings, or (3) misunderstanding of the computerized test instructions (Moser et al., 2011; Vaughan et al., 2014). Indeed, contrary to the screening measurements, specifically in the GG-FL_NE group, Figure 5 showed unexpectedly high phonological awareness scores at the first measurement point in a subset of the children, indicating some form of content-related support. Future studies which plan to implement computerized remote test sessions are recommended to make use of (1) parallel test versions to prevent practice effects and (2) remote supervisors using videoconferencing technology, an approach which has been investigated more often since the onset of the Covid-19 pandemic (Werfel et al., 2021) and which has already been proven feasible for assessing reading-related skills in young children (Eikerling et al., 2022). Nevertheless, we acknowledge that not conducting extra supervised phonological awareness tests at school forms a major flaw of the current study. Related to this limitation, given that we conducted a home-based intervention study, we could also not always exercise control over the intervention environment and more specifically, check whether children wore the headphones as instructed when playing the auditory intervention. Given that the story sound levels were calibrated through the internal tablet sound card over ATH M20x headphones, not (properly) wearing the headphone during the home-based story listening sessions could have possibly influenced the current intervention effects. The last limitation relates to the fact that we were unable to administer standardized reading and spelling assessments in kindergarten. Therefore, true effects of the auditory intervention on top of GG-FL on reading and spelling could not (yet) be measured. However, as we followed up our participants until the third grade, we will be able to investigate the long-term effects of both intervention programs on reading and writing in a future study. Moreover, the data log mechanisms implemented in GG-FL will also allow us to explore the predictive value of in-game measures on reading and writing ability in elementary grades, a possibility that has been widely considered as a major advantage of digital serious gaming interventions (Van Dijk et al., 2022).

Our findings support the Temporal Sampling Framework (Goswami, 2011) by showing a developmental speech-in-noise delay in pre-readers at cognitive risk for developmental dyslexia, which emerged during kindergarten, an important period for providing phonics-based preventive reading intervention. However, adding an extra auditory EE-intervention, which was shown to improve rise time sensitivity (Van Herck et al., 2021), on top of phonics-instruction in kindergarten did not boost speech-in-noise perception, phonological awareness, and letter knowledge in at-risk pre-readers. Future research however should investigate the potential of (1) including children with predefined deficits in auditory processing skills, (2) applying EE on child-directed speech, and (3) accelerating the timing of the story intervention in order to enhance auditory processing skills and prevent reading difficulties in children at cognitive risk.

## Data availability statement

The anonymized generated datasets that support findings for this study can be made available upon reasonable request. Interested parties seeking access to the data should contact the corresponding author explaining the specific purpose of their request.

## Ethics statement

The studies involving human participants were reviewed and approved by Medical Ethical Committee of University Hospital of Leuven, KU Leuven (B322201836276). Written informed consent to participate in this study was provided by the participants’ legal guardian/next of kin.

## Author contributions

FV collected and processed all data and wrote the original manuscript. ME and SV helped with the project administration, data collection, and processing, and reviewed and edited the draft. MV, JW, PG, and JV conceptualized and received funding for the project. Moreover, they coordinated the project and reviewed and edited the current draft. All authors contributed to the article and approved the submitted version.

## Funding

This study obtained funding from the Research Council of KU Leuven, Belgium (C14/17/046). JV was a postdoctoral researcher at KU Leuven and received a personal grant of the Research Foundation Flanders (FWO), Belgium (12T4818N).

## Conflict of interest

The authors declare that the research was conducted in the absence of any commercial or financial relationships that could be construed as a potential conflict of interest.

## Publisher’s note

All claims expressed in this article are solely those of the authors and do not necessarily represent those of their affiliated organizations, or those of the publisher, the editors and the reviewers. Any product that may be evaluated in this article, or claim that may be made by its manufacturer, is not guaranteed or endorsed by the publisher.
